# Traditional Chinese medicine Xiaosheng Powder for dry eye disease: A protocol for systematic review and meta analysis

**DOI:** 10.1097/MD.0000000000022019

**Published:** 2020-08-28

**Authors:** Jing Xu, Shuntai Chen, Xiaofeng Hao, Gaiping Wu, Shihui Wang, Hang Yuan, Qi Jin, Mei Sun, Like Xie

**Affiliations:** a Department of Ophthalmology, Eye Hospital; b Graduate School; c Department of Oncology, Guang’anmen Hospital, China Academy of Chinese Medical Sciences; d Graduate School, Beijing University of Chinese Medicine, Beijing, China.

**Keywords:** dry eye disease, protocol, randomized controlled trials, traditional Chinese medicine, Xiaosheng Powder

## Abstract

**Background:**

Dry eye disease (DED) has shown a significant increase in recent years, which seriously affects people's work and life. Xiaosheng Powder, a traditional Chinese medicine decoction, has been widely used in treating DED. However, there is no systematic review of the results of the study on this therapeutic effect. The purpose of this review is to evaluate the effectiveness and safety of Xiaosheng Powder in the treatment of DED.

**Methods and Analysis:**

The electronic databases to be searched will include MEDLINE (PubMed), Cochrane Central Register of Controlled Trials in the Cochrane Library, Excerpta Medica Database, China National Knowledge Infrastructure, China Scientific Journal Database, Wanfang Database and Chinese Biomedical Literature Database. Papers in English or Chinese published from inception to 2020 will be included without any restrictions. Improvement in Ocular Surface Disease Index will be assessed as the primary outcomes. Tear break-up time, Schirmer I test, fluorescent, adverse events, and the recurrence rate after at least 3 months of the treatment will be evaluated as secondary outcomes. We will conduct a meta-analysis of randomized controlled trial if possible. The methodological qualities, including the risk of bias, will be evaluated using the Cochrane risk of bias assessment tool, while confidence in the cumulative evidence will be evaluated using the Grading of Recommendations Assessment, Development, and Evaluation approach.

**Ethics and Dissemination:**

It is not necessary for a formal ethical approval because the data is not individualized. The results of this review will offer implications for the use of Xiaosheng Powder as a treatment for DED. This knowledge will inform recommendations by ophthalmologist and researchers who are interested in the treatment of DED. The findings of this systematic review will be disseminated through peer-reviewed publications and conference presentations.

**Trail registration number:**

PROSPERO CRD42020147709.

## 1 Introduction

### 1.1 Description of the condition

Dry eyedisease (DED) is a group of heterogeneous diseases composed of tear film dysfunction and ocular surface irritation symptoms, with diverse clinical manifestations.^[1]^ In 2017, Tear Film and Ocular Surface Society's Dry Eye Workshop II defined it as a multifactor disease of the ocular surface. It is characterized by the loss of steady state of the tear film, accompanied by ocular surface symptoms, and its pathogenesis includes tear film instability, high osmotic pressure of tears, ocular surface inflammation and injury, and neuroparesthesia.^[2,3]^ Common symptoms of DED include eye fatigue, foreign-body sensation, dry feeling, burning feeling, eye swelling, and pain,^[4,5]^ which affect people's life seriously. In recent years, the incidence of DED has been increasing year by year and developing toward the younger age. According to the epidemiological survey results, the incidence of DED is 5.5% to 33.7%,^[4]^ and more women than men.^[5]^

At present, the mainstream drug treatments commonly use local tears substitutes, anti-inflammatory and immunosuppressive drugs,^[6]^ hormones,^[7]^ and other drugs, but the effect of tears supplement for severe DED is poor. Long-term use of anti-inflammatory and immunosuppression and hormone replacement therapies can cause side effects. The drug therapy is not targeted at the cause of DED. And it can not alleviate the symptoms completely. And long-term using of eye drops aggravates the ocular surface injury. And surgical treatment^[8]^ of DED included lacrimal embolus implantation, lacrimal blocking, corneal conjunctiva transplantation,^[9]^ and submandibular gland transplantation.^[10]^ Patients are reluctant to receive such treatment.

DED was first recorded with “god water will be dry” in an ancient Chinese medical text “Standards of Diagnosis and Treatment” (Chinese name: “Zheng Zhi Zhun Sheng”). Based on the theory of traditional Chinese medicine (TCM), it is proposed that dry eye syndrome is closely related to the 5 internal organs and dysfunction. Among them, the liver is enlightened in the eye, the eye is outside the liver, and the liver is the main reservoir of blood, its fluid of tears moistens the eyes. If the blood in the liver is derelict, the secretion of tears is insufficient and eyes lost in moisture, resulting in DED. It can be seen that the cause of DED is closely related to the liver in TCM.

### 1.2 Description of the intervention

At present, TCM has a good effect on the treatment of DED. A number of clinical observation results suggest that the patients’ symptoms, physical signs, and laboratory indicators have improved.

Based on the basic theory of TCM, under the guidance of the holism concept of TCM and the theory of gasification, some scholars have found that “liver depression and Yin deficiency” is the etiology and pathogenesis of DED. And the function of Xiaosheng Powder with “Liver-soothing and Yin-nourishing” was used to treat DED.

### 1.3 How the intervention might work

Xiaosheng Powder is a combination of Xiaoyao Powder and Shengmai Decoction. Among them, Xiaoyao Powder is derived from “Prescriptions of the Bureau of Taiping People's Welfare Pharmacy” (Chinese name: “Taiping Huimin Heji Ju Fang”),^[11]^ which is a good medicine for soothing the liver and strengthening the spleen. Shengmai Decoction is the first prescription for nourishing yin and nourishing body fluid in clinic, which is derived from Li Dong-yuan's theory in “differentiation on endogenous and exogenous diseases” (Chinese name: “Neiwaishang Bianhuolun”).^[12]^ Xiaosheng Powder gathers the strengths of both sides and plays the role of soothing the liver, nourishing yin and moistening the eyes, so as to make the liver and Qi orderly, body fluid abundant, then the eyes can be nourished.

The main components of Xiaosheng Powder are Paeonia lactiflora, Bupleurum chinense, Rehmannia glutinosa, Chinses Angelica, Codonopsis pilosula, Ophiopogon japonicus, Schisandra chinensis, and Mentha haplocalyx Briq. The active ingredients of Paeonia lactiflora can regulate T and B lymphocyte. Moreover, it can also inhibit the over-expression of pain-related cytokines such as il-1, il-6, prostaglandin E, leukotriene B, and achieve anti-inflammatory effect.^[13]^ Bupleurum saikosaponins^[14]^ in Bupleurum chinense have effects on many inflammatory processes, including exudation and release of inflammatory mediators. Bupleurum saikosaponin have certain regulatory effects on specific immune function and nonspecific immune function of the body. Rehmannia glutinosa^[15]^ can significantly enhance the production of interleukin-2 (IL-2) in active lymphocytes and enhance the immune function. Chinses Angelica can promote the expression of IL-2, tumor necrosis factor γ, and other cytokines.^[16]^ Codonopsis pilosula can enhance the phagocytic activity of macrophages,^[17]^ enhancing the immune response of the whole body. Polysaccharide in Ophiopogon japonicus^[18]^ has immune activity, promotes humoral immunity and cellular immunity, induces a variety of cytokines, and improves the level of serum hemolysin antibody. Schisandra chinensis can increase immune suppression and promote lymphocyte transformation.^[19]^ The main active ingredient of Mentha haplocalyx Briq is menthol, which has obvious anti-inflammatory effect.^[20]^

Through immunohistochemical detection, it was found that Xiaosheng Powder had the function of anti-inflammatory factors production or release. Experimental studies have found that Xiaosheng Powder may reduce or inhibit the intensity of inflammatory response and the release of pro-inflammatory cytokines through immune regulation, so as to affect the apoptosis of ocular surface epithelial cells, lymphocytes, and lacrimal glands. And it prevents the apoptosis of goblet cells to exert its pharmacological effects, inhibiting or delaying the formation of dry eye.

### 1.4 Why it is important to do this review

So far, there is only no systematic review published investigating Xiaosheng Powder and DED. No definite conclusions on the effectiveness of Xiaosheng Powder for DED can be drawn. Using a stricter search strategy, a more objective outcome evaluation, and a rigorous review method, we expect that our systematic review will provide a more convincing conclusion.

### 1.5 Objectives

To evaluate the effectiveness and safety of Xiaosheng Powder for patients with DED systematically.

## 2 Methods and analysis

### 2.1 Study registration

**Figure 1:**
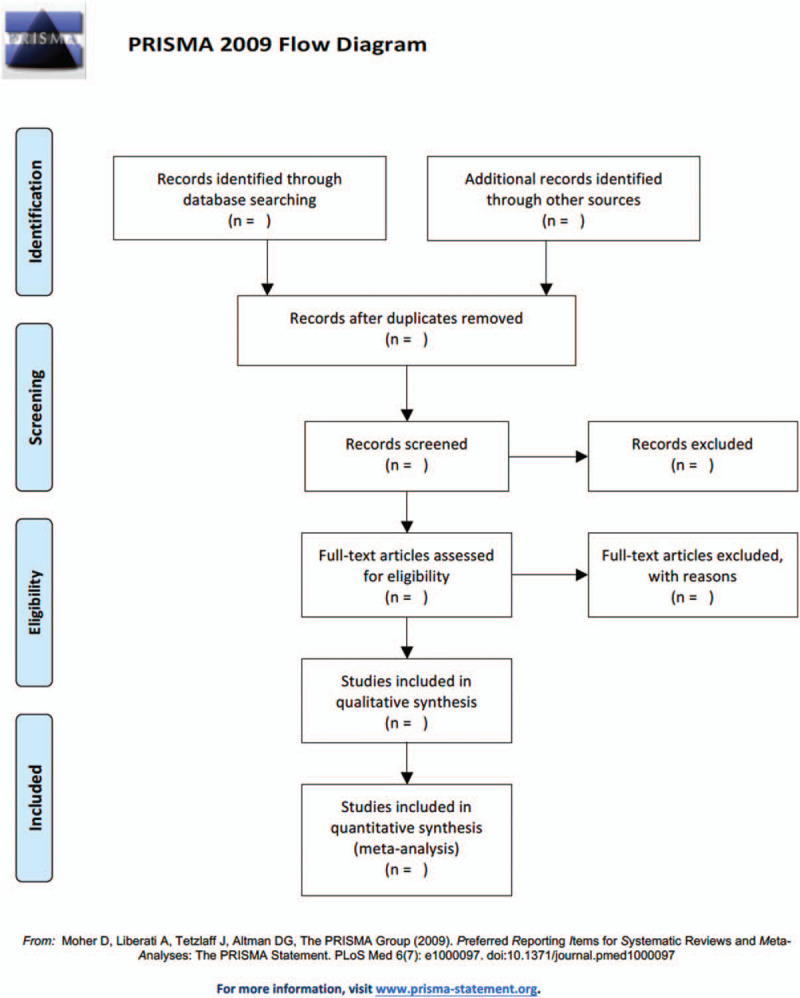
Flow diagram of studies search and selection.

This study will follow the guidelines outlined in the preferred reporting items for systematic reviews and meta-analysis (PRISMA) statement for meta-analyses of healthcare interventions.^[21]^ Additionally, the protocol adheres to the PRISMA Protocols.^[22]^ The selection process will be summarized according to PRISMA flow diagram (Fig. 1).

This review protocol is registered in the International Prospective Register of Systematic Reviews (PROSPERO CRD42020147709).

### 2.2 Inclusion criteria for study selection

#### 2.2.1 Types of studies

All randomized controlled trials regarding intervention of Xiaosheng Powder in DED will be included without restrictions on publication status.

#### 2.2.2 Types of participants

Patients diagnosed with DED will be included, regardless of their type, age, sex, and ethnicity.

#### 2.2.3 Types of interventions

Studies reporting Xiaosheng Powder treatment will be included. The control group will consist of patients given no treatment, placebo decoction, or other conventional treatments including local tears substitutes, anti-inflammatory and immunosuppressive drugs, hormones, and other drugs. Trials that evaluate the effects of Xiaosheng Powder used in combination with another therapy will also be included in the review.

#### 2.2.4 Types of outcome measures

##### 2.2.4.1 Primary outcomes

Improvement in symptoms, using ocular surface disease index, at least after 2 weeks of treatment.

##### 2.2.4.2 Secondary outcomes

Secondary outcomes include Tear break-up time, Schirmer I test , and fluorescent. The adverse events and recurrence rate after at least 3 months of the treatment will also be evaluated.

### 2.3 Search methods for identification of studies

#### 2.3.1 Electronic searches

The electronic databases to be searched will include MEDLINE (PubMed), Cochrane Central Register of Controlled Trials in the Cochrane Library, Excerpta Medica Database, China National Knowledge Infrastructure, China Scientific Journal Database, Wanfang Database and Chinese Biomedical Literature Database. Papers in English or Chinese published from inception to 2020 will be included without any restrictions. The search strategy for PubMed is shown in Table 1.

**Table 1:**
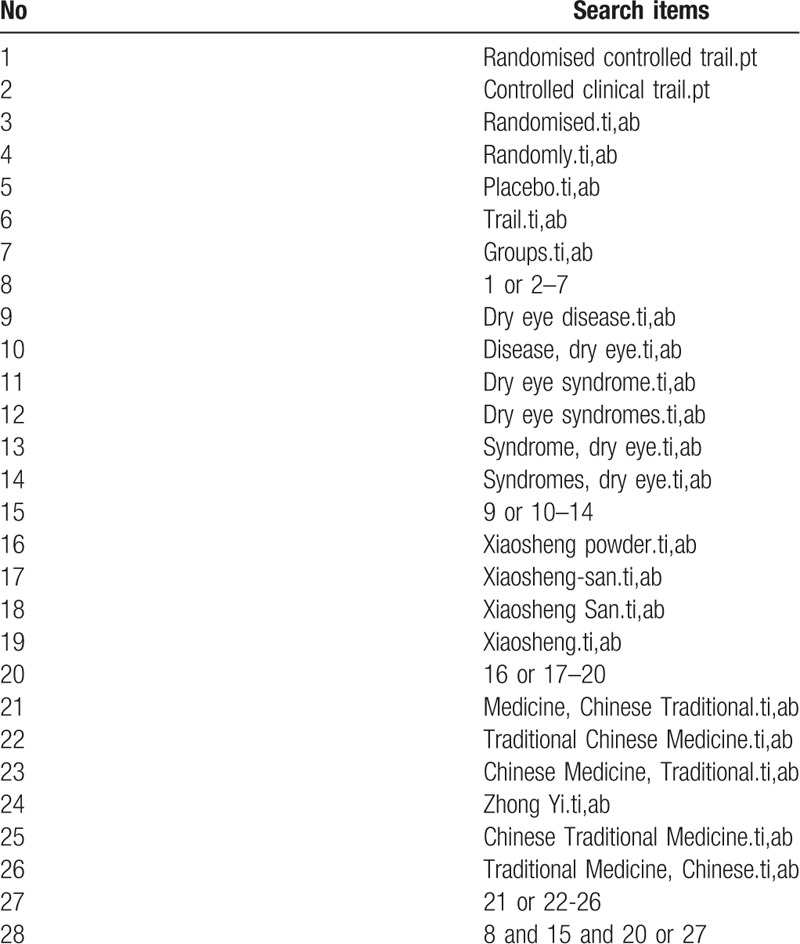
Search strategy used in PubMed database.

#### 2.3.2 Searching other resources

We will scan the reference lists of identified publications for additional trials. We will also search for other relevant systematic review articles and trials. The cited manuscripts will be manually searched once 1 systematic review is included.

### 2.4 Data collection and analysis

#### 2.4.1 Selection of studies

We plan to conduct this systematic review between January 1, 2021 and December 30, 2021. All reviewers have undergone training to ensure a basic understanding of the background and purpose of the review. The search results will be imported from the original databases to Endnote (V.X9.0). Two reviewers (JX and XH) will independently assess the eligibility of the retrieved studies according to the inclusion criteria. For preliminary study selection, only the title and abstract will be reviewed to exclude obviously inappropriate publications. Unmatched studies will be removed to a trash box in the software. The reasons for exclusion will be recorded as an Excel data set. The next step will be to further evaluate the included studies by reading their full-text version. The reference list will be checked by the 2 reviewers to identify potentially missing trials. The selection results will be cross-checked by the 2 reviewers. Any disagreement will be resolved by consensus. Any disagreements will be resolved by discussion between the 2 authors (JX and XH) and the third author (LX) for arbitration when necessary. We will contact the authors for further information when the reported data are not sufficient. Each eligible trial will be assigned a study ID formatted as follows: surname of the first author + space + year of publication (e.g., Yang 2017). The study flow diagram is shown in Figure 1.

#### 2.4.2 Data extraction and management

Two reviewers (JX and XH) will independently double-check the eligibility of the included studies and extract data by entering details into a predefined data acquisition form. We will obtain data for general information, participants, methods, interventions, outcomes, results, adverse events, conflicts of interest, ethical approval, and other information. Any discrepancy noticed in the process of data cross-checking will be resolved through discussion and the suggestion of a third reviewer (LX).

#### 2.4.3 Assessment of risk of bias in included studies

The risk of bias in included studies will be assessed by 2 independent reviewers (GW and SW) using the Cochrane Collaboration tool.^[23]^ We will evaluate the following domains for risk of bias as sequence generation, allocation sequence concealment, blinding of participants and personnel and outcome assessors, incomplete outcome data, selective outcome reporting, and other sources of bias. The assessments will be classified into 3 levels: low risk, high risk, and unclear risk. Unclear items in studies will be inquired by contacting corresponding authors for details. Any disagreement will be resolved by discussion with a third reviewer (LX).

#### 2.4.4 Measures of treatment effect

We will use RevMan V.5.3.5 software (The Cochrane Collaboration, Oxford, England) for data analysis and quantitative data synthesis.^[24]^ Dichotomous data will be analyzed by using a risk ratio with 95% confidence interval (95% CI).^[25]^ For continuous outcomes, data will be analyzed by using a mean difference or a standard mean difference with 95% CI.^[26,27]^

#### 2.4.5 Unit of analysis issues

The unit of analysis will be the individual participant. And the units of each outcome from different trials will be converted to the International System of Units before statistical analysis.

#### 2.4.6 Dealing with missing data

Corresponding authors will be connected by e-mail for detailed data if their studies’ information is not available. In case of no reply from the authors or contact person, we will impute the missing data with replacement values, treating these as if they were observed. The last observation carried forward imputation method will be used to assume a missing value and then an intention-to-treat analysis will be performed.^[28]^ Moreover, sensitivity analyses will be performed to assess how sensitive the results are to reasonable changes in the assumptions that are made. The potential impact of missing data on the final findings of the review will be discussed.

#### 2.4.7 Assessment of heterogeneity

χ^2^ test and I^2^ statistic test will be used to assess the heterogeneity. If the I^2^ value is <50% or *P* > .10, the study will not be considered to have heterogeneity and we will pool data using a fixed-effect model. Otherwise, significant statistic heterogeneity exists among the trial and meta-analysis will not be performed. χ^2^ test will be performed in the forest plot using RevMan V.5.3.5 to investigate the statistical heterogeneity and a *P* value of less than .10 will be considered significant, in line with the Cochrane Handbook.

#### 2.4.8 Assessment of reporting biases

Funnel plots will be used to detect reporting biases and small-study effects. If 10 or more studies are included in the meta-analysis, we will conduct a test for funnel plot asymmetry using the Egger method.^[29]^

#### 2.4.9 Data synthesis

Statistical analyses will be performed using RevMan manager V.5.3.5. If there is no substantial statistical heterogeneity (I^2^ < 50%), we will apply fixed effects model to perform in the analysis. The random-effects model will be used to combine the data if there is substantial statistical heterogeneity. If significant heterogeneity is detected, we will investigate possible reasons from both clinical and methodological perspectives, and provide a descriptive analysis or conduct subgroup analysis.

#### 2.4.10 Subgroup analysis

If the data are available and sufficient, we will also conduct subgroup analyses. Subgroup analysis will be conducted according to sex, age, and type of DED. Factors like different duration of Xiaosheng Powder therapies will also be taken into account.

#### 2.4.11 Sensitivity analysis

Sensitivity analysis will be used to verify the robustness of the review conclusions. We will consider several decision nodes within the process of the systematic review to implement a sensitivity review, such as sample size, the outcome of missing data, and methodological quality. Additionally, the analysis will be repeated after low-methodological-quality studies are excluded.

#### 2.4.12 Grading the quality of evidence

A summary of findings table will be generated and included in the final report. The quality of each selected studies will be evaluated through the Grading of Recommendations Assessment, Development and Evaluation approach by 3 investigators. The following domains will be assessed: risk of bias, consistency, directness, precision, publication bias and additional points. The assessments will be adjudicated into four levels: high, moderate, low, and very low quality.^[30]^

#### 2.4.13 Ethics and dissemination

Ethics approval will not be needed because date from individual patients will not be included and no privacy will be involved. We will disseminate the results of this systematic review by publishing the manuscript in a peer-reviewed journal or presenting the findings at a relevant conference.

## 3 Discussion

TCM has unique advantages in the treatment of DED, and is widely used in China to improve the relevant symptoms. This systematic review will provide an assessment of the current state of Xiaosheng Powder treatment for DED. And currently no systematic review related to Xiaosheng Powder for DED has been published in English. The process of conducting this review will be divided into 4 parts: identification, study inclusion, data extraction, and data synthesis. This review has some potential limitations, especially language barriers. Only studies published in Chinese and English will be included. And the different criteria for efficacy evaluation and forms of DED may cause significant heterogeneity in this review. High-quality trials may be deficient to generate convincing conclusions.

### 3.1 Strengths and limitations of this study

This systematic review will comprehensively assess the efficacy and safety of Xiaosheng Powder for the treatment of DED.

Our review may provide evidence for researchers and be helpful for clinical practitioners in treating DED.

Due to language barriers, only studies published in Chinese and English will be included. And the different criteria for efficacy evaluation and forms of DED may cause significant heterogeneity in this review. High-quality trials may be deficient to generate convincing conclusions.

## Author contributions

LX had the concept for the study. JX, SC, and XH developed and conducted the search strategy. The manuscript of the protocol was drafted by JX and revised by XH, QJ, HY, and MS. JX and SC will independently screen the potential studies and extract data from the included studies. GW and SW will assess the risk of bias and finish data synthesis. XL will arbitrate any disagreements and ensure that no errors occur during the review. All authors read, provided feedback, and approved the final manuscript.
